# Psychometric properties of the European health literacy survey questionnaire short-short form (HLS-EU-Q6) among Slovenian youth, adults, and older adults

**DOI:** 10.1186/s12889-026-28926-5

**Published:** 2026-10-07

**Authors:** Andrej Kirbiš, Stefani Branilović, Mitja Vrdelja, Monika Lamot

**Affiliations:** 1 Department of Sociology, Faculty of Arts, University of Maribor, Koroška Cesta 160, 2000 Maribor, Slovenia; 2 National Institute of Public Health, Trubarjeva, Trubarjeva cesta 2, Ljubljana, 1000 Slovenia

**Keywords:** HLS-EU-Q6, Validity, Reliability, Age groups, Vaccine attitudes, Slovenia

## Abstract

**Background:**

Health literacy is an important determinant of health behaviours and self-rated health, making brief, valid, and reliable assessment tools essential. The six-item European Health Literacy Survey Questionnaire (HLS-EU-Q6) has rarely been validated across age groups and has not been validated in Slovenia. This study evaluated its validity and reliability among youth, adults, and older adults.

**Methods:**

Data were drawn from the Health Literacy of Adults in Slovenia survey (HLS-SI19; *N* = 3,360), including youth (18–30 years), adults (31–64 years), and older adults (65 + years). Construct validity was assessed using confirmatory factor analysis and Item Response Theory (Samejima's Graded Response Model); convergent, discriminant, concurrent, concurrent-predictive, and predictive (criterion-related) validity, together with internal reliability, were assessed using correlations and hierarchical regression analyses.

**Results:**

A one-factor structure demonstrated good fit among older adults but weaker fit among youth, adults, and the total sample. IRT analyses showed satisfactory item discrimination and measurement precision, although four items showed age-related differential item functioning, indicating partial measurement invariance across age groups. Internal consistency was satisfactory across all groups (Cronbach's α = 0.76–0.84). Discriminant validity relative to communicative and navigational health literacy, and concurrent validity with the HLS-EU-Q47 (r = 0.892), were supported in all age groups. Predictive validity appeared stronger among adults and older adults than among youth: among younger participants health literacy predicted only self-rated health, whereas among adults and older adults it also predicted more favourable attitudes toward vaccination.

**Conclusions:**

The Slovenian HLS-EU-Q6 is a reliable and practical screening instrument, particularly for adults and older adults, although its construct and predictive validity among youth warrant further age-sensitive refinement.

**Supplementary Information:**

The online version contains supplementary material available at https://doi.org/10.1186/s12889-026-28926-5.

## Introduction

Health literacy refers to the capacities of individuals to deal with the demands of health [1]. The World Health Organization defines it as “the ability of individuals to gain access to, understand and use information in ways which promote and maintain good health for themselves, their families and their communities” [2]. Health literacy is a multidimensional concept that has been operationalised through a range of questionnaires and scales. Some instruments are designed for specific populations,the Risk Estimate of Inadequate Health Literacy (REIHL), for instance, was developed as a practitioner-friendly screener for older adults [3]. Other established measures include the Rapid Estimate of Adult Literacy in Medicine (REALM) and the Test of Functional Health Literacy in Adults (TOFHLA) [4],however, one of the most widely used instruments in recent research is the HLS-EU questionnaire [5].

The HLS-EU questionnaire was developed to capture the multidimensionality of health literacy. In its original form it comprises 47 items addressing three health domains (healthcare, disease prevention, and health promotion) and four cognitive information-processing competencies (finding, understanding, appraising, and using information), each rated on a four-point scale ranging from very easy to very difficult [1]. Because of its length, shorter versions were subsequently developed comprising 16 items [6–8], 12 items [9–11], and 6 items [12–14], which have been validated in various national contexts.

Valid and reliable health literacy instruments are essential, because health literacy is consistently associated with health outcomes [15, 16]. People with limited health literacy are less likely to engage in disease prevention, are less responsive to health education, report poorer overall health and poorer understanding of their treatment, and are more likely to be hospitalised [4, 16, 17]. Understanding individuals’ health literacy therefore allows health-related information, services, and education programmes to be tailored so as to improve health literacy itself [4].

Achieving this goal requires valid and reliable measurement, yet the evidence base for the HLS-EU-Q6 has two limitations that matter for how the instrument is used. First, although the questionnaire has been validated in several languages [7, 13, 14, 18], these validations typically rely on a single pooled adult sample and report only construct validity and internal reliability. It remains unclear whether the HLS-EU-Q6 functions adequately across the lifespan, and in particular whether it performs well among youth and older adults, the two groups whose health literacy and information-processing differ most from the adult norm. Second, few studies have tested whether the HLS-EU-Q6 is associated with health behaviours and self-rated health, even though such predictive validity is precisely what justifies using a six-item screener in population surveys and clinical practice.

The present study addresses both gaps using a large, representative national sample (*N* = 3,360). We evaluate the full validity spectrum of the Slovenian HLS-EU-Q6, including its predictive (i.e., criterion-related) validity for self-rated health, health behaviours, and vaccine attitudes, and we assess its psychometric performance separately among youth (18–30 years), adults (31–64 years), and older adults (65 years and older). We include vaccine attitudes as an additional, policy-relevant criterion because health literacy is a known correlate of vaccination attitudes and uptake, which makes it a meaningful test of whether a brief screener captures clinically relevant variation [17, 19, 20]. This design moves beyond a narrow population gap: establishing whether a brief screener performs well for older adults but poorly for youth helps identify the age groups for which the HLS-EU-Q6 may provide the most appropriate psychometric performance.

### Health literacy measures across age groups

When assessing the psychometric properties of the HLS-EU questionnaire, it is important to consider health literacy differences across age groups. There is evidence of lower health literacy among older individuals, attributed partly to declining cognitive ability and health impairments [21]. A German study of people older than 65 years found that all respondents had limited health literacy, with especially low levels among those above 76 years, and that limited health literacy was associated with financial deprivation and limited functional health literacy [22]. A study across eight European countries similarly found limited health literacy to be concentrated among older people and among those who were less educated, financially deprived, or of lower socioeconomic status [23]. Berens and colleagues [21], in a study of 2,000 participants aged 15 to 65 and older, likewise found that health literacy decreased with age and that limited health literacy was associated with limited functional health literacy, more frequent doctor visits, and lower social status.

The psychometric properties of health literacy measures may also vary across age groups owing to developmental, cognitive, and experiential differences. Younger individuals (≤ 30 years) often display health information-seeking behaviours and cognitive processing styles that differ from those of middle-aged adults (31–64 years) and older adults (≥ 65 years). Older adults, for instance, may face declining cognitive function and more frequent healthcare interactions, both of which can affect their health literacy and how they interpret health information [24]. A Chinese study that adapted the Chinese Resident Health Literacy Scale (CRHLS) simplified the original instrument because it was more time-consuming for older adults than for younger respondents, particularly those with lower literacy, and because older respondents struggled with items outside their knowledge or interest, such as understanding drug labels or describing liver function [25]. Younger populations, such as adolescents, are also reported to have difficulty understanding existing health literacy items, owing to their more limited experience with the health system, which may translate into lower measured health literacy and signals a need for age-appropriate measurement [26]. The digital divide further shapes health literacy: younger people are generally more adept with digital health resources, whereas older adults may have more limited access or skills [27]. Extending these comparisons to include young people is therefore essential for understanding age-related differences and ensuring the validity of health literacy measures across the lifespan.

Because health literacy differs between younger and older individuals, several studies have validated HLS-EU questionnaires across age groups. Such studies have typically focused on adults and older adults [24], with little attention to younger populations [27]. Lamot and Kirbiš [6], for example, analysed a representative sample of Slovenian adults and older adults and found adequate validity and reliability of the HLS-EU-Q16 in both groups. Rouquette et al. [7] reported differential item functioning by age in the French HLS-EU-Q16, with two items, interpreting a doctor's advice and judging the need for a second opinion, proving more challenging for older respondents than for younger ones. A Korean validation of the HLS-EU-Q47 among 254 older adults confirmed the questionnaire’s reliability and validity and likewise found that health literacy decreased with age [28]. Conversely, Sukys and colleagues [29] assessed the HLS-EU-Q47 among 912 university students, confirming good psychometric properties overall but finding that younger individuals had difficulty accessing health information within the health promotion domain and applying it within the disease prevention domain. These findings echo those of Domanska et al. [30], who reported that adolescents were unfamiliar with some HLS-EU-Q47 items because of limited experience with certain health-related tasks and disease prevention.

Age-group disparities in health literacy, and in the psychometric properties of the longer HLS-EU forms, underscore the need to examine whether the HLS-EU-Q6 maintains a consistent factor structure and adequate discriminant and predictive validity across age cohorts. Ensuring that the instrument functions equivalently across age groups is crucial for accurate assessment and for tailoring public health interventions. Without such validation, health literacy levels may be misinterpreted, potentially leading to ineffective or misdirected health policies and programmes. A thorough investigation of the psychometric properties of the HLS-EU-Q6 across age groups is therefore essential to establish its reliability and validity in diverse demographic contexts.

### Psychometric properties of HLS-EU-Q6 worldwide

As noted above, no study has examined the psychometric properties of the HLS-EU-Q6 in a Slovenian sample. Lamot and Kirbiš [6] found that the 16-item questionnaire showed adequate validity and reliability and was therefore appropriate for measuring health literacy in Slovenia, but they did not assess the HLS-EU-Q6. Outside Slovenia, several studies have used the HLS-EU-Q6 and evaluated its validity [14, 18, 31]. Bergman and colleagues [14] evaluated the psychometric properties – including floor and ceiling effects, validity, reliability, and response patterns – of the Arabic HLS-EU-Q16 and HLS-EU-Q6 in Sweden. They found no floor or ceiling effects for either questionnaire, indicating balanced response distributions, and both showed construct validity in four of five expected correlations, as well as good structural validity and reliability. However, the HLS-EU-Q6 showed limited criterion validity, suggesting that it may not align well with the longer versions.

Another study assessed the psychometric properties of both short forms against the HLS-EU-Q47 in a Brazilian Portuguese sample. Strong correlations were found between the indices, all exceeding 0.8, and Cronbach’s alpha exceeded 0.7 for both the HLS-EU-Q16 and the HLS-EU-Q6, reflecting acceptable reliability. The three questionnaires nonetheless yielded different health literacy levels, with the HLS-EU-Q47 and HLS-EU-Q16 producing the most similar distributions; the authors concluded that the 16-item form showed strong internal consistency and close alignment with the validated HLS-EU-Q47 [18].

Lorini and colleagues [13] validated the Italian HLS-EU-Q16 and HLS-EU-Q6 and found evidence of reliability and validity for both. Both questionnaires were normally distributed, showed strong correlations, and had Cronbach’s alpha values above 0.70. In terms of health literacy levels, the HLS-EU-Q6 classified 8.9% of respondents as having inadequate, 66.5% as problematic, and 24.6% as sufficient health literacy, while the HLS-EU-Q16 yielded a broadly similar distribution (11.8% inadequate, 55.2% problematic, and 33% sufficient). Both questionnaires were significantly associated with education, training or employment in healthcare, and self-perceived health status; the only difference was that the 16-item form was significantly associated with long-term illness, whereas the HLS-EU-Q6 was not.

Mousum and colleagues [32] compared the HLS-EU-Q16 and HLS-EU-Q6 using national survey data from Bangladesh, applying descriptive statistics, Cronbach’s alpha, confirmatory factor analysis, and chi-square tests. Both instruments showed good psychometric properties and strong internal consistency (Cronbach’s alpha above 0.85). The HLS-EU-Q16 displayed a three-factor structure with a second-order general component, while the HLS-EU-Q6 fit a three-factor model with solid fit indices. The authors concluded that both questionnaires are valid and reliable measures of health literacy but recommended the 16-item form because of its stronger psychometric properties.

In contrast, Rouquette and colleagues [7] found acceptable psychometric properties for the French HLS-EU-Q16 but did not find support for the HLS-EU-Q6. Despite high Cronbach’s alpha coefficients (both above 0.80) and a high Spearman correlation between the two forms – indicating that they measure the same construct – agreement between the health literacy levels they classified was poor. Convergent and discriminant validity for the 6-item form were also unconvincing, with a kappa coefficient of only 0.06. The one-factor model for the HLS-EU-Q6 showed generally acceptable CFI and TLI but a high RMSEA, indicating room for improvement, and the second-order model could not be evaluated reliably owing to computational issues.

### Hypotheses

Based on this review of the literature, we developed the following hypotheses.

- H1: The HLS-EU-Q6 demonstrates a one-factor solution among the general population (H1a), youth (H1b), adults (H1c), and older adults (H1d).
- H2: The HLS-EU-Q6 shows discriminant validity compared to communicative and navigational health literacy measures among the general population (H2a), youth (H2b), adults (H2c), and older adults (H2d).
- H3: The HLS-EU-Q6 demonstrates concurrent-predictive validity among the general population (H3a), youth (H3b), adults (H3c), and older adults (H3d).
- H4: When adjusting for sociodemographic and socioeconomic confounders, the HLS-EU-Q6 demonstrates predictive (criterion-related) validity for health behaviours, self-rated health and vaccine attitudes among the general population (H4a), youth (H4b), adults (H4c), and older adults (H4d).

## Methods

### Sample

This study used data from the Health Literacy of Adults in Slovenia survey (HLS-SI19), which comprised 3,360 participants. Women made up 53.7% of the sample, and participants ranged in age from 18 to 99 years (M = 52.6). Data were collected through computer-assisted personal interviews (CAPI), self-administered questionnaires (SAQ), and computer-assisted web interviews (CAWI). Participants were selected using multi-stage random sampling and had to be at least 18 years old to be included [33]. The following three subsections describe the three age groups: youth (18–30 years), adults (31–64 years), and older adults (65 years and older).

#### Young

The youth group comprised 495 participants aged 18 to 30 years (M = 24.6 years), 47.1% of whom were women. Most had completed secondary technical education (27.3%), followed by gymnasium (21.8%), a master’s-level university degree (11.9%), higher professional or bachelor’s education (11.7%), lower or secondary vocational education (10.7%), and primary education (5.7%); 3.2% held a master of science or doctorate, 0.2% a specialisation, and 0.2% had no formal education. Half were employed (50.3%) and a third were students (33.1%). Their mean self-assessed socioeconomic status was mid-scale (M = 5.69, SD = 1.56).

#### Adults

The adult group comprised 1,989 participants aged 31 to 64 years (M = 48.6 years) [34], 54.4% of whom were women. Their mean self-assessed socioeconomic status was mid-scale (M = 5.46, SD = 1.61). Secondary technical education was the most common level (25.2%), followed by lower or secondary vocational education (17.1%). Most were employed (65.1%), with smaller proportions retired (15.1%), unemployed (8.2%), self-employed (7.3%), doing household work (2%), or unable to work (1.5%).

#### Older adults

The older adult group comprised 876 participants aged 65 years and older (M = 73.9 years) [34], 55.7% of whom were women. Their mean self-assessed socioeconomic status was the lowest of the three groups (M = 5.18, SD = 1.56). Almost all were retired (96.3%). The most common educational levels were secondary technical education (24.4%), lower or secondary vocational education (23.4%), and primary education (24%).

### Measures

#### Health literacy questionnaire

We used the Slovenian version of the HLS-EU-Q47 to validate the HLS-EU-Q6. The six items were selected on the basis of previous literature [14, 18, 31] and were: “Assess when you need a second opinion from another doctor?”,“Use the information that your doctor gives you to make decisions about your illness.”; “Find information on how to deal with mental health problems, such as stress or depression.”; “Assess whether the information on health risks available in the media is reliable? (e.g., TV, internet, or other means of communication)”; “Find information about activities that are good for your mental well-being? (e.g., meditation, exercise, walking, pilates)”; and “Understand the information available in the media on how to stay healthier? (e.g., internet, newspapers, magazines)”. All items were rated on a four-point scale (1 = very difficult; 4 = very easy). For both the HLS-EU-Q47 and the HLS-EU-Q6 we computed summed scores.

#### Communicative health literacy

As a separate construct for the discriminant validity analyses, we measured communicative health literacy using 11 items rated on a four-point scale (1 = very difficult; 4 = very easy). Example items include: “How easy or difficult is it to get the information you need from your doctor?”; “…to ask your doctor questions during a consultation?”; and “…to be involved in decisions about your health during a conversation with your doctor?” [33]. The scale showed excellent internal consistency (Cronbach’s alpha = 0.93 for youth, 0.94 for adults, and 0.95 for older adults). We computed a summed score from these 11 items for the validity analyses.

#### Navigational health literacy

Similarly, we measured navigational health literacy, a distinct construct for the discriminant validity analyses, using 12 items rated on a four-point scale (1 = very difficult; 4 = very easy). Example items include: “How easy or difficult is it to choose a particular health service (e.g., to choose between different hospitals)?”; “…to find information about the quality of a particular health service?”; and “…to judge whether a particular health service will meet your expectations?” [33], [35]. This scale also showed excellent internal consistency (Cronbach’s alpha = 0.93 for youth, 0.93 for adults, and 0.94 for older adults).

#### Self-rated health, health behaviours and vaccine attitudes

To measure self-rated health we used the question “What is your general state of health?”, originally rated on a five-point scale (1 = very good; 5 = very poor) and recoded so that higher values indicate better health (1 = very poor to 5 = very good).

For health behaviours we used four questions: “In a typical week, how many days do you smoke any tobacco products?”; “…how many days do you drink beverages containing alcohol (e.g., beer, wine, spirits, cocktails, ready-made mixed alcoholic drinks, liqueurs, or home-made alcoholic drinks)?”; “…how many days have you been physically active for a total of 30 min or more, with at least a slight increase in breathing or heart rate (e.g., sport or other leisure activity, work, household or garden chores, or active travel)?”; and “…how many days do you eat fruit, vegetables, or salad (excluding potatoes, freshly squeezed juices, and juice concentrates)?”. Responses were recoded to range from 0 (never) to 8 (seven days).

Following Lubej and Kirbiš [17] vaccine attitudes were measured with three items assessing perceived importance, effectiveness, and safety of vaccines (“Vaccinations are important to protect myself and my children,” “Overall I think vaccinations are effective,” and “Overall I think vaccinations are safe”), rated on a four-point Likert scale (1 = strongly agree, 4 = strongly disagree) [33]. Responses were reverse-coded so that higher scores reflected more favourable attitudes toward vaccination. The three items were then summed to create a composite vaccine attitudes score (Cronbach’s alpha = 0.91 for youth, 0.90 for adults, and 0.87 for older adults).

#### Demographic and socioeconomic predictors

As demographic and socioeconomic predictors we used gender (1 = male, 2 = female), age (18 to 99 years), education (1 = no formal education to 11 = master’s degree or doctorate), self-assessed socioeconomic status (1 = lowest to 10 = highest position in society), and self-assessed economic deprivation, measured with the question “How easily can you pay your living expenses each month?” on a four-point scale (1 = very easily; 4 = very difficult).

### Statistical analyses

Analyses were conducted in SPSS 29 and R. The analytic plan followed the validation logic used by Pelikan et al. [10] and Lamot and Kirbiš [6], but was adapted to the HLS-EU-Q6 and to the comparison of youth, adults, and older adults. We first tested the expected one-factor solution with confirmatory factor analysis using maximum likelihood estimation. Model fit was evaluated with χ^2^, CFI, TLI, RMSEA, and SRMR. CFI and TLI values of at least 0.90 were interpreted as acceptable and values of at least 0.95 as good, while RMSEA and SRMR values below 0.08 were treated as acceptable.

To complement the Classical Test Theory analyses, we additionally evaluated the HLS-EU-Q6 using Item Response Theory in R with the *mirt* package. Because all six HLS-EU-Q6 items are ordered polytomous indicators with four response categories, a one-dimensional Samejima’s Graded Response Model was estimated using the expectation–maximization algorithm. Item discrimination and category-threshold parameters were obtained for all items. Overall model fit was evaluated using the M2 statistic together with RMSEA, SRMSR, CFI, and TLI. Item Information Functions and the Test Information Function were examined to assess measurement precision across the latent health literacy continuum, and marginal reliability was estimated within the IRT framework.

To investigate whether the instrument functioned equivalently across age groups, multigroup graded response models were estimated for youth, adults, and older adults. Youth served as the reference group, with its latent mean fixed to 0 and its latent variance fixed to 1; latent means and variances were freely estimated for adults and older adults. Differential Item Functioning was evaluated using likelihood-ratio tests for discrimination and threshold parameters. Threshold differences were interpreted as uniform DIF, whereas differences in discrimination were interpreted as non-uniform DIF. P-values were adjusted using the Benjamini–Hochberg procedure. Because several items initially demonstrated DIF, a sequential DIF procedure using the sample-size-adjusted Bayesian information criterion was additionally applied to identify a robust set of DIF items.

A final partial-invariance model was then estimated in which the four items identified as exhibiting robust DIF were allowed to vary across age groups, while the two items not retained as exhibiting robust DIF following the sequential DIF procedure, using information provided by a doctor to make decisions about illness and understanding media information on how to stay healthier, were retained as invariant anchor items. This partial-invariance model was compared with the fully invariant model using a likelihood-ratio test and information criteria.

Convergent validity and reliability were then assessed from standardized factor loadings, average variance extracted (AVE > 0.50), composite reliability (CR > 0.70), and Cronbach's alpha (α > 0.70). Discriminant validity was examined by correlating the HLS-EU-Q6 with communicative and navigational health literacy, while concurrent validity was assessed through its association with the full HLS-EU-Q47. To evaluate criterion-related evidence, we estimated two sets of regression models. Linear regression models tested whether sociodemographic and socioeconomic predictors were associated with HLS-EU-Q6 scores, following Pelikan et al. [10] and Lamot and Kirbiš [6]. Hierarchical regression models then tested whether the HLS-EU-Q6 added explanatory value for self-rated health, health behaviours, and vaccine attitudes. Because all variables were measured simultaneously, the regression analyses provide evidence of criterion-related validity in a cross-sectional context. We retain the term "predictive validity" throughout the manuscript to remain consistent with previous HLS-EU validation studies (e.g., [6, 10], while acknowledging that no temporal prediction can be inferred.

## Results

### Construct validity

First, we assessed the construct validity of the HLS-EU-Q6 using confirmatory factor analysis (CFA). We tested the one-factor structure of the HLS-EU-Q6 using maximum likelihood estimation. Results for the whole sample and each age group are presented in Table 1. Chi-square was significant for the whole sample (χ^2^ = 391.371, *p* < 0.01), youth (χ^2^ = 69.772, *p* < 0.01), adults (χ^2^ = 286.278, *p* < 0.01), and older adults (χ^2^ = 65.739, *p* < 0.01), indicating some misfit between model and data. Because chi-square is sensitive to sample size, we also examined other fit indices.

**Table 1:** Construct validity of the HLS-EU-Q6

	**Whole sample**	**Young people**	**Adults**	**Older adults**
χ^2^ (df)	391.371**	69.772**	286.278**	65.739**
RMSEA	0.115**	0.118**	0.127**	0.089**
CFI	0.928	0.903	0.903	0.965
TLI	0.881	0.838	0.838	0.942
SRMR	0.045	0.053	0.053	0.033

*CFI* Comparative Fit Index, *TLI* Tucker-Lewis Index, *RMSEA* Root Mean Square Error of Approximation, *SRMR* Standardized Root Mean Square Residual. ** *p* < 0.01

The RMSEA exceeded the recommended threshold of 0.08 for the whole sample (0.115), youth (0.118), and adults (0.127), indicating poor fit; only older adults approached an acceptable value (0.089). The CFI exceeded 0.95 for older adults (0.965), indicating good fit, but was suboptimal for youth and adults (0.903 for both). Similarly, the TLI was acceptable only for older adults (0.942) and considerably lower for youth and adults (0.838 for both). The SRMR was within the acceptable range for all groups, lowest for older adults (0.033), followed by the whole sample (0.045), youth, and adults (both 0.053).

Based on these findings, H1 is partially confirmed. The one-factor solution is supported for older adults (H1d), where the model showed acceptable fit on all indices except the marginal RMSEA. However, H1a (general population), H1b (youth), and H1c (adults) are not confirmed, as the one-factor structure showed significant misfit, particularly on RMSEA and TLI, despite acceptable CFI and SRMR. The one-factor model thus appears to represent the construct well for older adults but not for the general population, youth, or adults.

We also assessed the internal consistency of the HLS-EU-Q6. For the full sample, Cronbach’s alpha was 0.803, indicating good internal consistency. The three age groups also showed good internal consistency, with Cronbach’s alpha values of 0.76 for youth, 0.78 for adults, and 0.84 for older adults.

### Item response theory and differential item functioning

To complement the CTT analyses, a graded response model (GRM) was estimated. Results are reported in Tables S1–S4 and Figures S1 and S2 in the Supplementary Material. All six items demonstrated moderate to high discrimination (a = 1.45–2.55), indicating that each item effectively differentiated respondents across the latent health literacy continuum. Item discrimination was highest for the items "Find information on how to deal with mental health problems, such as stress or depression" (a = 2.55) and "Find information about activities that are good for your mental well-being" (a = 2.22), whereas the item "Assess when you need a second opinion from another doctor" showed the lowest, yet still satisfactory, discrimination (a = 1.45). Threshold parameters were ordered for every item (b1 < b2 < b3), indicating appropriate functioning of the response categories. Consistent with these findings, the Item Information Functions (Figure S2, Supplementary Material) showed that individual items differed in the amount of measurement information they provided across the latent health literacy continuum. Items with higher discrimination parameters generally yielded greater information and therefore measured respondents with higher precision across the corresponding ranges of the latent trait.

Differential Item Functioning (DIF) analyses indicated that four of the six items demonstrated robust age-related DIF following sequential purification: "Assess when you need a second opinion from another doctor", "Find information on how to deal with mental health problems, such as stress or depression", "Assess whether the information on health risks available in the media is reliable", and "Find information about activities that are good for your mental well-being." In contrast, the item "Use the information that your doctor gives you to make decisions about your illness" showed no evidence of DIF across any analysis, whereas "Understand the information available in the media on how to stay healthier" demonstrated significant omnibus DIF but was not retained in the sequential DIF solution. Uniform DIF was observed for five items, suggesting age-related differences in category thresholds, whereas non-uniform DIF was identified for three items: "Find information on how to deal with mental health problems, such as stress or depression", "Assess whether the information on health risks available in the media is reliable", and "Find information about activities that are good for your mental well-being"—indicating age-related differences in item discrimination.

Inspection of the item parameters showed that discrimination generally increased with age for the four robust DIF items. For example, discrimination for the item "Find information about activities that are good for your mental well-being" increased from a = 1.60 among youth to a = 2.59 among older adults, indicating stronger differentiation among older respondents. Threshold parameters also varied across age groups, suggesting that respondents of different ages required somewhat different levels of latent health literacy to endorse equivalent response categories for these items.

Marginal reliability remained high across all age groups, ranging from 0.789 among youth to 0.844 among older adults. Measurement precision was highest among older adults, who achieved the greatest peak information (7.31) and the widest interval of reliable measurement toward higher levels of the latent trait. In contrast, the instrument showed slightly lower precision among youth while remaining adequately reliable across a broad portion of the health literacy continuum.

Figure S1 presents the Test Information Functions across the three age groups. Overall, the HLS-EU-Q6 demonstrated adequate measurement precision across a broad range of the latent health literacy continuum in all groups. However, measurement precision differed somewhat across age categories. Older adults exhibited the highest peak information (7.31 at θ = −2.00), followed by adults (5.41 at θ = −2.45) and youth (4.65 at θ = −2.40). Consequently, conditional standard errors were lowest among older adults (SE = 0.37), indicating the greatest measurement precision within this subgroup. Across all age groups, the instrument was most informative at below-average levels of health literacy, with precision gradually declining toward higher levels of the latent trait. The range of reliable measurement (conditional reliability ≥ 0.70) extended from θ = −3.55 to 1.55 among adults, from −3.30 to 1.30 among youth, and from −2.95 to 2.25 among older adults. These findings indicate that the HLS-EU-Q6 provides reliable assessment across a wide portion of the health literacy continuum while showing only modest differences in measurement precision between age groups.

Finally, releasing the four DIF items significantly improved model fit (Δχ^2^(32) = 191.34, *p* < 0.001), indicating that allowing these parameters to vary across age groups provided a better representation of the data, supporting partial rather than full measurement invariance across age groups. Importantly, however, the latent mean differences remained virtually unchanged (Youth = 0.00, Adults = −0.27, Older adults = −0.85), indicating that the observed age differences in health literacy cannot be explained solely by age-related item bias.

The IRT analyses confirmed that the HLS-EU-Q6 demonstrates good item discrimination and satisfactory measurement precision across the health literacy continuum. Although several items exhibited age-related differential item functioning, latent mean differences remained unchanged after accounting for DIF, indicating that the observed age-group differences primarily reflect genuine differences in health literacy rather than measurement bias.

### Discriminant validity

Following the approach Pelikan et al. [10] applied to the HLS19-Q12, we examined how strongly the HLS-EU-Q6 related to two distinct but related constructs, communicative and navigational health literacy, in the overall sample and within each age group (Table 2). In the full sample, the HLS-EU-Q6 correlated moderately with communicative health literacy (r = 0.486, *p* < 0.01) and more strongly with navigational health literacy (r = 0.655, *p* < 0.01). The same pattern held within age groups: correlations with communicative health literacy were moderate (youth r = 0.413, adults r = 0.456, older adults r = 0.546,all *p* < 0.01), and correlations with navigational health literacy were higher (youth r = 0.587, adults r = 0.626, older adults r = 0.698; all *p* < 0.01). Because communicative and navigational health literacy are related but conceptually distinct constructs, these moderate correlations indicate that the HLS-EU-Q6 is associated with, but not redundant to, them. On this basis we consider H2 supported across the general population (H2a), youth (H2b), adults (H2c), and older adults (H2d), while noting that the relatively high correlation with navigational health literacy among older adults (r = 0.698) points to more limited separation in that group.

**Table 2:** Discriminant validity of the HLS-EU-Q6 across sample and age groups

	Whole sample		Young people		Adults		Older adults	
	1	2	1	2	1	2	1	2
1. HLS-EU-Q6	-	-	-	-	-	-	-	-
2. COM_HL	0.486**	-	0.413**	-	0.456**	-	0.546**	-
3. NAV_HL	0.655**	0.525**	0.587**	0.545**	0.626**	0.488**	0.698**	0.557**

Values are Pearson correlations; column 1 reports correlations with the HLS-EU-Q6 and column 2 with communicative health literacy. ** *p* < 0.01. *COM_HL* communicative health literacy, *NAV_HL* navigational health literacy

### Concurrent-predictive validity

To assess concurrent-predictive validity, we conducted linear regression analyses with gender, age, education, economic deprivation, and self-assessed socioeconomic status as predictors. In the whole sample, all predictors had a statistically significant effect on health literacy (Table 3). Among youth, only gender and economic deprivation were significant. Among adults, all predictors except age were significant. The directions of the significant effects were as expected. Among adults, for example, higher HLS-EU-Q6 scores were associated with being female (β = 0.105, *p* < 0.01), higher education (β = 0.076, *p* < 0.05), lower economic deprivation (β = −0.170, *p* < 0.01), and higher socioeconomic status (β = 0.106, *p* < 0.01).

**Table 3:** Concurrent-predictive validity of the HLS-EU-Q6 across sample and age groups

	Whole sample			Young people			Adults			Older adults		
	β	S.E	p	β	S.E	p	β	S.E	p	β	S.E	p
Gender	0.093	0.110	<.001	0.169	0.268	<.001	0.105	0.125	<.001	0.077	0.207	0.014
Age	−0.182	0.003	<.001	0.072	0.037	0.134	−0.022	0.007	0.327	−0.287	0.015	<.001
Education	0.098	0.027	<.001	−0.002	0.076	0.96	0.076	0.034	0.003	0.152	0.062	<.001
ED	−0.16	0.087	<.001	−0.126	0.199	0.012	−0.17	0.11	<.001	−0.208	0.187	<.001
SES	0.087	0.039	<.001	0.078	0.095	0.124	0.106	0.049	<.001	0.041	0.082	0.296

R^2^ young people = 0.065; R^2^ adults = 0.098, R^2^ older adults = 0.191. Standardized beta coefficients are reported. *S.E* standard error, *ED* Economic deprivation, *SES* Socioeconomic status

Table 3 also presents the concurrent-predictive validity of the HLS-EU-Q6 among older adults. In this group, almost all predictors had a statistically significant effect on the HLS-EU-Q6 except socioeconomic status. Unlike among adults, age had a significant negative effect among older adults, indicating that younger older adults had higher health literacy (β = −0.287, *p* < 0.01). These findings support H3 for the general population (H3a), adults (H3c), and older adults (H3d), and provide partial support among youth (H3b), where only gender and economic deprivation were significant predictors. Overall, concurrent-predictive validity appeared to be stronger among Slovenian adults and older adults.

### Concurrent validity

Concurrent validity was assessed by correlating the HLS-EU-Q6 with the original HLS-EU-Q47; the correlation was strong and significant (r = 0.892, *p* < 0.01). Concurrent validity was strong across all three age groups, and strongest among older adults (r = 0.893, *p* < 0.01), followed by adults (r = 0.884, *p* < 0.01) and youth (r = 0.878, *p* < 0.01). The HLS-EU-Q6 thus demonstrates consistently strong concurrent validity across age groups in the Slovenian sample.

### Predictive (criterion-related) validity

To evaluate predictive validity, we conducted hierarchical regression analyses examining the association between the HLS-EU-Q6 and health outcomes. Table 4 presents the results for the general population. After adjusting for sociodemographic and socioeconomic confounders, the HLS-EU-Q6 was associated with self-rated health (β = 0.137, *p* < 0.01), lower alcohol consumption (β = −0.052, *p* < 0.01), greater physical activity (β = 0.121, *p* < 0.01), and higher fruit and vegetable intake (β = 0.045, *p* < 0.05). No significant relationship was observed with smoking. Including the HLS-EU-Q6 increased the explained variance in the four models in which it was a significant predictor, although these increments were generally modest across all analyses.

**Table 4:** Predictive (criterion-related) validity of the HLS-EU-Q6

	Self-rated health		Smoking		Alcohol consumption		Physical activity		Fruit/vegetable intake	
	Step 1	Step 2	Step 1	Step 2	Step 1	Step 2	Step 1	Step 2	Step 1	Step 2
Gender	−0.025	−0.038*	−0.063**	−0.065**	−0.307**	−0.302**	−0.042*	−0.053**	0.147**	0.142**
Age	−0.388**	−0.363**	−0.178**	−0.173**	0.049**	0.040*	0.003	0.025	0.172**	0.180**
Education	0.069**	0.055**	−0.120**	−0.123**	0.06**	0.066**	−0.047*	−0.059**	−0.015	−0.02
ED	−0.172**	−0.150**	0.071**	0.075**	−0.032	−0.04	−0.025	−0.006	−0.067**	−0.060**
SES	0.108**	0.097**	−0.013	−0.015	−0.002	0.003	0.04	0.03	0.095**	0.091**
HLS-EU-Q6		0.137**		0.026		−0.052**		0.121**		0.045*
R^2^	0.274	0.29	0.049	0.049	0.098	0.1	0.005	0.018	0.066	0.066
F	246.344**	222.241**	33.519**	28.279**	70.748**	60.470**	3.477**	9.860**	46.005**	39.437**
ΔR^2^		0.016		0.001		0.002		0.013		0.002
ΔF		74.140**		2.024		8.286**		41.558**		6.227*

^**^p < 0.01; * *p* < 0.05. *ED* Economic deprivation, *SES* Socioeconomic status. Reported are standardized coefficients

Table 5 presents the results for youth. The HLS-EU-Q6 significantly predicted only self-rated health (β = 0.134, *p* < 0.01), adding a small but significant increment to the explained variance (ΔR^2^ = 0.017, ΔF = 8.416, *p* < 0.01) beyond sociodemographic and socioeconomic characteristics. It showed no significant association with smoking, alcohol consumption, physical activity, or fruit and vegetable intake.

**Table 5:** Predictive (criterion-related) validity of the HLS-EU-Q6 in the youth population

	Self-rated health		Smoking		Alcohol consumption		Physical activity		Fruit and vegetable intake	
	Step 1	Step 2	Step 1	Step 2	Step 1	Step 2	Step 1	Step 2	Step 1	Step 2
Gender	−0.075	−0.098*	−0.038	−0.031	−0.226**	−0.229**	−0.086	−0.100*	0.151**	0.139**
Age	−0.031	−0.041	0.167**	0.170**	0.099*	0.097*	0.055	0.049	0.039	0.034
Education	−0.001	0.000	−0.280**	−0.281**	0.025	0.025	−0.028	−0.027	0.008	0.009
ED	−0.129*	−0.112*	0.094	0.089	−0.037	−0.034	−0.036	−0.026	−0.045	−0.036
SES	0.110*	0.099*	0.002	0.005	0.001	0.000	0.08	0.073	0.143**	0.138**
HLS-EU-Q6		0.134**		−0.042		0.020		0.080		0.070
R^2^	0.047	0.064	0.095	0.097	0.062	0.063	0.019	0.025	0.057	0.062
F	4.609**	5.304**	9.801**	8.306**	6.205**	5.193**	1.813	1.99	5.643**	5.098**
ΔR^2^		0.017		0.002		0		0.006		0.005
ΔF		8.416**		0.847		0.189		2.838		2.297

^**^p < 0.01; * *p* < 0.05. *ED* Economic deprivation, *SES* Socioeconomic status. Reported are standardized coefficients

Table 6 summarises the results for adults, among whom the HLS-EU-Q6 predicted self-rated health (β = 0.123, *p* < 0.01), greater physical activity (β = 0.048, *p* < 0.05), and lower alcohol consumption (β = −0.054, *p* < 0.05). It significantly increased the explained variance in all three models (ΔR^2^ = 0.002 to 0.014), indicating incremental predictive value for multiple health behaviours.

**Table 6:** Predictive (criterion-related) validity of the HLS-EU-Q6 in the adult population

	Self-rated health		Smoking		Alcohol consumption		Physical activity		Fruit and vegetable intake	
	Step 1	Step 2	Step 1	Step 2	Step 1	Step 2	Step 1	Step 2	Step 1	Step 2
Gender	−0.019	−0.032	−0.046*	−0.047*	−0.300**	−0.294**	−0.036	−0.041	0.145**	0.142**
Age	−0.202**	−0.200**	−0.067**	−0.067*	0.028	0.027	0.065**	0.066**	0.176**	0.177**
Education	0.084**	0.075**	−0.144**	−0.145**	0.054*	0.058*	−0.060*	−0.064*	0.003	0
ED	−0.199**	−0.178**	0.077**	0.079**	−0.016	−0.025	−0.031	−0.022	−0.085**	−0.079**
SES	0.115**	0.102**	−0.003	−0.004	−0.01	−0.004	0.016	0.011	0.081**	0.077**
HLS-EU-Q6		0.123**		0.011		−0.054*		0.048*		0.033
R^2^	0.176	0.19	0.037	0.037	0.09	0.092	0.011	0.013	0.066	0.067
F	83.098**	75.793**	14.795**	12.360**	38.050**	32.701**	4.160**	4.146**	27.435**	23.204**
ΔR^2^		0.014		0		0.003		0.002		0.001
ΔF		32.526**		0.215		5.515*		4.044*		1.98

^**^p < 0.01; * *p* < 0.05. *ED* Economic deprivation, *SES* Socioeconomic status. Reported are standardized coefficients

Table 7 presents the results for older adults, for whom the HLS-EU-Q6 predicted self-rated health (β = 0.178, *p* < 0.01), lower alcohol consumption (β = −0.110, *p* < 0.01), and greater physical activity (β = 0.191, *p* < 0.01). The corresponding gains in explained variance were statistically significant across all three outcomes (self-rated health ΔR^2^ = 0.026, ΔF = 26.261, *p* < 0.01; alcohol consumption ΔR^2^ = 0.010, ΔF = 9.630, *p* < 0.05; physical activity ΔR^2^ = 0.029, ΔF = 27.415, *p* < 0.01).

**Table 7:** Predictive (criterion-related) validity of the HLS-EU-Q6 in the older adult population

	Self-rated health		Smoking		Alcohol consumption		Physical activity		Fruit and vegetable intake	
	Step 1	Step 2	Step 1	Step 2	Step 1	Step 2	Step 1	Step 2	Step 1	Step 2
Gender	−0.004	−0.018	−0.079*	−0.079*	−0.348**	−0.339**	−0.013	−0.027	0.153**	0.152**
Age	−0.200**	−0.149**	−0.203**	−0.201**	−0.026	−0.057	−0.245**	−0.191**	−0.082*	−0.077*
Education	0.075*	0.048	0.000	−0.001	0.038	0.055	0.001	−0.028	−0.03	−0.033
ED	−0.205**	−0.168**	0.020	0.021	−0.064	−0.087*	−0.047	−0.007	−0.074	−0.071
SES	0.110*	0.103**	−0.067	−0.067	0.014	0.018	0.043	0.035	0.073	0.073
HLS-EU-Q6		0.178**		0.004		−0.110**		0.191**		0.016
R^2^	0.149	0.175	0.053	0.053	0.142	0.152	0.069	0.098	0.04	0.04
F	29.541**	29.732**	9.443**	7.862**	27.826**	25.032**	12.385**	15.214**	6.973**	5.835**
ΔR^2^		0.026		0		0.01		0.029		0
ΔF		26.261**		0.013		9.630*		27.415**		0.179

^**^p < 0.01; * *p* < 0.05. *ED* Economic deprivation, *SES* Socioeconomic status. Reported are standardized coefficients

Finally, in Table 8 we present the results for vaccine attitudes as the outcome. In the whole sample, the HLS-EU-Q6 was positively associated with more favourable attitudes toward vaccination (β = 0.142, *p* < 0.01). The inclusion of the HLS-EU-Q6 significantly increased the explained variance from R^2^ = 0.046 to R^2^ = 0.063 (ΔR^2^ = 0.018, ΔF = 57.287, *p* < 0.01), indicating that health literacy contributes to the prediction of vaccine attitudes beyond sociodemographic characteristics. When the analyses were conducted separately by age group, the HLS-EU-Q6 was not associated with vaccine attitudes among young people (β = 0.085, p > 0.05), and the increase in explained variance was not statistically significant (ΔR^2^ = 0.007, ΔF = 3.412, p > 0.05). In contrast, significant positive associations were observed among adults (β = 0.161, *p* < 0.01) and older adults (β = 0.196, *p* < 0.01). Adding the HLS-EU-Q6 significantly improved the explanatory power of the models in both groups, increasing the explained variance by 2.3% among adults (ΔR^2^ = 0.023, ΔF = 46.128, *p* < 0.01) and by 3.2% among older adults (ΔR^2^ = 0.032, ΔF = 26.346, *p* < 0.01). These increases were consistent across age groups, supporting the incremental contribution of health literacy to explaining vaccine attitudes. Taken together, these findings provide partial support for H4. The HLS-EU-Q6 demonstrated predictive validity in the general population (H4a), among adults (H4c), and among older adults (H4d), but only limited predictive validity among young people (H4b), for whom it significantly predicted self-rated health but not health behaviours or vaccine attitudes.

**Table 8:** Predictive (criterion-related) validity of the HLS-EU-Q6; outcome: vaccine attitudes

	Whole sample		Young people		Adults		Older adults	
	Step 1	Step 2	Step 1	Step 2	Step 1	Step 2	Step 1	Step 2
Gender	0.002	−0.011	−0.081	−0.095*	0.013	−0.004	0.01	−0.005
Age	0.028	0.052**	−0.200**	−0.206**	0.043	0.046	0.018	0.071
Education	0.111**	0.099**	0.199**	0.199**	0.143**	0.132**	0.091*	0.065
ED	−0.131**	−0.108**	−0.142**	−0.131**	−0.124**	−0.095**	−0.137**	−0.097*
SES	0.03	0.019	0.071	0.065	0.023	0.007	−0.005	−0.010
HLS-EU-Q6		0.142**		0.085		0.161**		0.196**
R^2^	0.046	0.063	0.098	0.105	0.053	0.076	0.035	0.068
F	29.164**	34.300**	9.837**	8.810**	20.343**	25.059**	5.573**	9.189**
ΔR^2^		0.018		0.007		0.023		0.032
ΔF		57.287**		3.412		46.128**		26.346**

^**^p < 0.01; * *p* < 0.05. *ED* Economic deprivation, *SES* Socioeconomic status. Reported are standardized coefficients

The predictive (criterion-related) validity of the HLS-EU-Q6 was therefore not uniform across age groups. Among youth it was limited, significantly predicting only self-rated health and not health behaviours or vaccine attitudes. Among adults and older adults, the HLS-EU-Q6 showed more satisfactory predictive validity, significantly predicting self-rated health, lower alcohol consumption, physical activity, and vaccine attitudes in both groups. Even so, it was not associated with all examined outcomes, as no significant associations were observed for smoking or fruit and vegetable intake among different age groups, indicating that its predictive validity is partial rather than comprehensive.

## Discussion

This study investigated the validity and reliability of the European Health Literacy Survey Questionnaire short-short form (HLS-EU-Q6) among Slovenian youth, adults, and older adults. Previous validations of the HLS-EU-Q6 in different countries have shown mixed results: some language versions demonstrated adequate psychometric properties [13, 18], while others did not support its validity [7, 14].

Regarding the construct validity, the one-factor solution performed best among older adults: CFI and SRMR were acceptable, TLI was above 0.90, and RMSEA was only slightly above the recommended threshold. Fit was weaker among youth and adults, where RMSEA exceeded the recommended threshold and TLI was below 0.90 despite CFI values around the acceptable 0.90 level and acceptable SRMR. This pattern partly mirrors previous HLS-EU-Q6 validations: Bergman et al. [14] reported support for a one-factor solution, whereas Rouquette et al. [7] found that the one-factor HLS-EU-Q6 did not meet recommended fit thresholds. The comparison with the Slovenian HLS-EU-Q16 validation is informative. Lamot and Kirbiš [6] found that the theoretically proposed HLS-EU-Q16 structure did not fit Slovenian adults and older adults well in CFA, but exploratory analyses suggested a modified three-factor structure among adults and a two-factor structure among older adults. These findings suggest that, although the HLS-EU-Q6 can be used as a brief one-factor screening instrument, its factorial structure may not fully capture the complexity of health literacy among younger and middle-aged adults.

The Item Response Theory analyses strengthened the psychometric evaluation of the Slovenian HLS-EU-Q6 by providing item-level evidence that complements the CFA results. All six items demonstrated satisfactory discrimination and appropriately ordered threshold parameters, indicating that they effectively differentiated respondents across the latent health literacy continuum and that the response categories functioned as intended. The Item and Test Information Functions further showed that the questionnaire provided adequate measurement precision across a broad range of health literacy levels in all three age groups, although measurement precision was highest among older adults. These findings support the suitability of the HLS-EU-Q6 as a brief screening instrument across the lifespan while also demonstrating that some items contribute more information than others.

The DIF analyses also provide important practical information regarding the use of the HLS-EU-Q6 across age groups. Although four items demonstrated age-related differential functioning, releasing these items substantially improved model fit while latent mean differences remained largely unchanged. This indicates that the observed age differences in health literacy primarily reflect genuine differences in the underlying construct rather than measurement artefacts. Consequently, although complete measurement invariance was not supported, the HLS-EU-Q6 retains sufficient comparability across age groups for most research applications when partial invariance is acknowledged.

To assess discriminant validity, we correlated the HLS-EU-Q6 with communicative and navigational health literacy. Discriminant validity was confirmed in all three age groups, with good results for communicative and strong results for navigational health literacy. Pelikan et al. [10] and Lamot and Kirbiš [6] similarly confirmed discriminant validity using these scales, although on different instruments. Pelikan et al. [10] assessed the 12-item questionnaire and Lamot and Kirbiš [6] the 16-item questionnaire.

For concurrent-predictive validity, all sociodemographic and socioeconomic predictors had a significant effect on the HLS-EU-Q6 in the whole sample. Across age groups, however, only gender and economic deprivation remained significant among youth; among adults, all predictors except age were significant; and among older adults, only self-assessed socioeconomic status was non-significant. The HLS-EU-Q6 therefore suggests somewhat stronger concurrent-predictive validity among adults and older adults than among youth, although these differences were not formally tested. Lorini et al. [13] likewise found, using chi-square tests, that the HLS-EU-Q6 was associated with educational level and with training or employment in healthcare. More broadly, health literacy is shaped by sociodemographic and socioeconomic factors: women tend to report lower rates of various diseases and healthier lifestyles than men [36, 37],older individuals tend to have poorer overall health and more limited health literacy than younger adults [38],and lower education, economic deprivation, and lower socioeconomic status are associated with more limited health literacy [39, 40].

Concurrent validity was excellent in the whole sample and in each age group. This is consistent with Pedro et al. [18], who also reported high correlation coefficients among the HLS-EU-PT, HLS-EU-PT-16, HLS-EU-PT-Q6, G-HL16, and G-HL6. By contrast, Rouquette et al. [7] assessed concurrent validity by correlating the HLS-EU-Q6 with the Functional, Communicative and Critical Health Literacy (FCCHL) scale and found a poor correlation, indicating that the French HLS-EU-Q6 did not demonstrate concurrent validity.

Finally, we examined predictive (criterion-related) validity using hierarchical regression analyses with the HLS-EU-Q6 as a predictor of self-rated health, several health behaviours and vaccine attitudes. In the overall sample, higher HLS-EU-Q6 scores were associated with better self-rated health, lower alcohol consumption, more frequent physical activity, higher fruit and vegetable intake and more favourable vaccine attitudes. Across age groups, however, the HLS-EU-Q6 was associated only with self-rated health among youth, and self-rated health, lower alcohol consumption, more frequent physical activity, and favourable vaccine attitudes among adults and older adults. The pattern of findings suggests somewhat stronger criterion-related validity among adults and older adults than among youth, although this validity remained limited, as it did not significantly predict two of the five health outcomes (smoking and fruit and vegetable intake). Our overall-sample results are consistent with studies showing that higher health literacy is associated with better self-rated health and healthier behaviours. Aaby et al. [41], for example, found that higher health literacy was associated with more frequent physical activity, a healthier diet, and less smoking, and a study of older Chinese adults found that those with higher health literacy were less likely to smoke or drink alcohol, exercised more, and reported better self-rated health [42]. The differences between youth on the one hand and adults and older adults on the other may reflect evidence that younger people have lower health literacy because of unfamiliarity with some health domains, and that lower health literacy is associated with unhealthier diets and greater engagement in risky behaviours, such as substance use over time [30, 43]. However, although the HLS-EU-Q6 significantly contributed to the prediction of several outcomes, the increases in explained variance were generally modest, indicating that health literacy represents one of several determinants of these health-related outcomes rather than a dominant predictor.

The significant association between health literacy and vaccine attitudes among adults and older adults is also consistent with previous research showing that individuals with higher health literacy are more likely to hold positive attitudes towards vaccination and to accept recommended vaccines [17, 19, 20]. Health literacy may thus develop over the life course: adults with higher health literacy are more likely to engage in preventive behaviours such as physical activity and healthy eating [44], while older adults with higher health literacy are more likely to manage their medications effectively and engage in health-promoting activities [45].

Overall, the Slovenian HLS-EU-Q6 demonstrated generally satisfactory psychometric properties, albeit with some limitations. Discriminant and concurrent validity were supported in the overall sample and across all age groups. Concurrent-predictive and predictive (criterion-related) validity appeared stronger among adults and older adults than among youth, although these observations are based on descriptive comparisons across age groups and should therefore be interpreted cautiously. The multigroup IRT analyses further supported the overall robustness of the instrument by showing that the observed age differences largely reflected genuine differences in health literacy rather than measurement bias, despite evidence of partial measurement invariance across age groups.

Several limitations should be noted. First, the study was based on cross-sectional data, which precludes causal inference and does not allow temporal prediction of subsequent health outcomes. Consequently, although we use the term predictive validity to remain consistent with previous HLS-EU validation studies [6, 10], the present findings should be interpreted as evidence of criterion-related validity obtained within a cross-sectional design. Longitudinal studies are needed to establish whether HLS-EU-Q6 scores predict future health behaviours and health outcomes over time. Second, the HLS-EU-Q6 did not significantly predict smoking or fruit and vegetable intake, suggesting that other determinants may drive these behaviours. Third, although the IRT analyses supported the overall psychometric adequacy of the HLS-EU-Q6, several items exhibited age-related Differential Item Functioning, indicating partial rather than full measurement invariance across the lifespan. Although accounting for DIF did not alter the substantive age differences observed in this study, future research should continue to examine age-specific item functioning and evaluate whether alternative wording or age-sensitive scoring could further improve comparability across age groups. Fourth, the weaker performance of the HLS-EU-Q6 in some other studies, such as the French validation, highlights potential challenges in cross-cultural adaptation and applicability. Finally, the short form may still fail to fully capture the multidimensionality of health literacy.

Future studies should also examine whether age-sensitive scoring, additional short-form items, or complementary measures improve the assessment of health literacy across the lifespan. Further work should also test the predictive validity of the HLS-EU-Q6 for diet, physical activity, smoking, and vaccination outcomes in longitudinal designs.

Nevertheless, because the HLS-EU-Q6 requires only six items while demonstrating satisfactory psychometric performance, it represents a practical screening tool for large epidemiological surveys and routine public health monitoring where questionnaire length is constrained, particularly among adults and older adults. Our findings also point to a need for tailored health literacy interventions, especially for younger people, who show lower health literacy, and underline the importance of integrating health literacy education into school curricula and public health campaigns to address developmental gaps in younger populations.

## Conclusion

The HLS-EU-Q6 demonstrated overall good psychometric properties and proved to be a valid and practical instrument for assessing health literacy among Slovenian youth, adults, and older adults. Complementing the Classical Test Theory analyses with Item Response Theory provided additional evidence that the instrument possesses satisfactory item discrimination and good measurement precision across the latent health literacy continuum. Although several items exhibited age-related Differential Item Functioning, accounting for this partial measurement non-invariance did not substantially alter the observed age differences in health literacy, indicating that these differences primarily reflect genuine variation in the underlying construct rather than measurement bias.

Among adults and older adults, the HLS-EU-Q6 was associated with self-rated health, several health behaviours, and more favourable vaccine attitudes, whereas its predictive (criterion-related) validity was more limited among youth and for certain health behaviours. Overall, these findings support the HLS-EU-Q6 as a satisfactory brief screening instrument for use in population-based research and public health practice, particularly among adults and older adults. Future research should further examine age-sensitive item functioning, evaluate the stability of the identified Differential Item Functioning across different cultural and population contexts, and investigate the longitudinal relationship between health literacy and subsequent health outcomes.

## Supplementary Information

Supplementary Material 1.

## Data Availability

The data that support the findings of this study are available from the authors upon reasonable request.
