# Supplementary material for: Psychometric properties of the European health literacy survey questionnaire short-short form (HLS-EU-Q6) among Slovenian youth, adults, and older adults
Source: BMC Public Health. 2026 Oct 7;26:3052. doi: 10.1186/s12889-026-28926-5 (PMC13692978; doi:10.1186/s12889-026-28926-5)
Supplement: Supplementary file 1 [file 12889_2026_28926_MOESM1_ESM.docx]

Table S1 Graded response model item parameters for the HLS-EU-Q6

| **Item** | **a** | **b1** | **b2** | **b3** |
| --- | --- | --- | --- | --- |
| Assess when you need a second opinion from another doctor? | 1.449 | -2.93 | -0.704 | 1.758 |
| Use the information that your doctor gives you to make decisions about your illness | 1.75 | -3.285 | -1.83 | 1.079 |
| Find information on how to deal with mental health problems, such as stress or depression | 2.548 | -2.356 | -1.038 | 0.834 |
| Assess whether the information on health risks available in the media is reliable? (e.g., TV, internet, or other means of communication) | 1.565 | -2.315 | -0.36 | 1.635 |
| Find information about activities that are good for your mental well-being? (e.g., meditation, exercise, walking, pilates) | 2.219 | -2.777 | -1.597 | 0.485 |
| Understand the information available in the media on how to stay healthier? (e.g., internet, newspapers, magazines) | 2.015 | -2.35 | -0.997 | 1.151 |

*Note.* *a* = discrimination parameter. *b1–b3* = category thresholds estimated under the graded response model.

Table S2 Differential item functioning across age groups

| **Item** | **Omnibus χ²** | **df** | **BH p** | **Uniform DIF** | **Nonuniform DIF** | **Sequential DIF** |
| --- | --- | --- | --- | --- | --- | --- |
| Assess when you need a second opinion from another doctor? | 49.98 | 8 | <.001 | Yes | No | Yes |
| Use the information that your doctor gives you to make decisions about your illness | 11.4 | 8 | 0.18 | No | No | No |
| Find information on how to deal with mental health problems, such as stress or depression | 40.43 | 8 | <.001 | Yes | Yes | Yes |
| Assess whether the information on health risks available in the media is reliable? (e.g., TV, internet, or other means of communication) | 41.36 | 8 | <.001 | Yes | Yes | Yes |
| Find information about activities that are good for your mental well-being? (e.g., meditation, exercise, walking, pilates) | 65.1 | 8 | <.001 | Yes | Yes | Yes |
| Understand the information available in the media on how to stay healthier? (e.g., internet, newspapers, magazines) | 25.64 | 8 | 0.001 | Yes | No | No |

*Note.* Omnibus DIF tested differences in both discrimination and thresholds. Uniform DIF refers to threshold differences only, whereas nonuniform DIF refers to discrimination differences. Sequential DIF represents the final robust DIF solution after iterative purification. P-values were adjusted using the Benjamini–Hochberg procedure.

Table S3 Item parameters of DIF items by age group

| **Age group** | **Item** | **a** | **b1** | **b2** | **b3** |
| --- | --- | --- | --- | --- | --- |
| Youth | Assess when you need a second opinion from another doctor? | 1.248 | -3.191 | -0.619 | 1.784 |
| Adults |  | 1.384 | -3.598 | -1.142 | 1.442 |
| Older adults |  | 1.689 | -3.159 | -1.298 | 1.079 |
| Youth | Find information on how to deal with mental health problems, such as stress or depression | 2.122 | -2.37 | -1.084 | 0.652 |
| Adults |  | 2.457 | -2.857 | -1.508 | 0.462 |
| Older adults |  | 2.839 | -2.904 | -1.532 | 0.396 |
| Youth | Assess whether the information on health risks available in the media is reliable? (e.g., TV, internet, or other means of communication) | 1.51 | -2.453 | -0.49 | 1.349 |
| Adults |  | 1.522 | -2.63 | -0.728 | 1.298 |
| Older adults |  | 1.716 | -2.999 | -0.934 | 1.194 |
| Youth | Find information about activities that are good for your mental well-being? (e.g., meditation, exercise, walking, pilates) | 1.602 | -3.545 | -2.079 | 0.208 |
| Adults |  | 2.039 | -3.51 | -2.273 | 0.076 |
| Older adults |  | 2.586 | -3.056 | -1.841 | 0.173 |

*Note*. Parameter estimates are reported from the final partial invariance model.

Table S4 Latent health literacy distribution and measurement precision across age groups

| **Age group** | **Latent mean** | **Marginal reliability** | **Maximum information** | **Reliable θ range** |
| --- | --- | --- | --- | --- |
| Youth | 0 | 0.789 | 4.65 | −3.30–1.30 |
| Adults | −0.272 | 0.803 | 5.41 | −3.55–1.55 |
| Older adults | −0.853 | 0.844 | 7.31 | −2.95–2.25 |

*Note*. Maximum information represents the highest value of the test information function.

Figure S1 Test information curve for the HLS-EU-Q6 across age groups

*Note*. Test information functions were estimated separately for youth (18–30 years), adults (31–64 years), and older adults (65 years and older). Greater information values indicate higher reliability (lower conditional standard error) at each level of the latent health literacy trait (θ).

Figure S2 Item information functions for the six HLS-EU-Q6 items

*Note*. Item Information Functions estimated using Samejima's graded response model. Higher information values indicate greater measurement precision and lower conditional standard error at a given level of the latent health literacy trait (θ). Items with higher discrimination parameters contribute greater information across the latent continuum.
